# Exosomes in Bone Cancer: Unveiling their Vital Role in Diagnosis, Prognosis, and Therapeutic Advancements

**DOI:** 10.7150/jca.95709

**Published:** 2024-06-03

**Authors:** Subhrojyoti Ghosh, Atharva Anand Mahajan, Anuvab Dey, Ramya Lakshmi Rajendran, Ankita Chowdhury, Sushmita Sen, Subhobrata Paul, Sourav Majhi, Chae Moon Hong, Prakash Gangadaran, Byeong-Cheol Ahn, Anand Krishnan

**Affiliations:** 1 Department of Biotechnology, Indian Institute of Technology Madras, Chennai, Tamil Nadu 600036, India.; 2 Advanced Centre for Treatment, Research, and Education in Cancer, Tata Memorial Centre, Mumbai, Maharashtra 410210, India.; 3 Department of Biosciences and Bioengineering, Indian Institute of Technology Guwahati, North Guwahati, Assam 781039, India.; 4 Department of Nuclear Medicine, School of Medicine, Kyungpook National University, Daegu 41944, Republic of Korea.; 5 Department of Biochemical Engineering and Biotechnology, Indian Institute of Technology, Delhi 110016, India.; 6 Department of Chemical Pathology, School of Pathology, Faculty of Health Sciences, University of the Free State, Bloemfontein, 9300, South Africa.; 7 Department of Nuclear Medicine, Kyungpook National University Hospital, Daegu 41944, Republic of Korea.; 8 BK21 FOUR KNU Convergence Educational Program of Biomedical Sciences for Creative Future Talents, Department of Biomedical Science, School of Medicine, Kyungpook National University, Daegu 41944, Korea.

**Keywords:** Exosomes, Bone cancer, Tumor microenvironment, Biomarkers, Liquid biopsy, Precision medicine, Extracellular vesicles

## Abstract

Bone cancer among adolescents and children exhibits varying survival outcomes based on disease state. While localized bone cancer cases have a survival rate exceeding 70%, metastatic, refractory, and recurrent forms are associated with significantly poorer prognoses. Initially believed to be mere vehicles for cellular waste disposal, exosomes are now recognized as extracellular vesicles facilitating intercellular communication. These vesicles influence cellular behaviors by transporting various biomolecules, such as proteins, DNA, RNA, and lipids, among cells. The role of exosomes in regulating the progression of bone cancer is increasingly evident, impacting critical processes like tumorigenesis, proliferation, metastasis, angiogenesis, immune evasion, and drug resistance. Current research underscores the substantial potential of exosomes in promoting the progression and development of bone cancer. This review delves into the complex process of exosome biogenesis, the variety of cell-derived exosome sources, and their applications in drug delivery and therapeutics. It also examines ongoing clinical trials focused on exosome cargo levels and discusses the challenges and future directions in exosome research. Unlike costly and invasive traditional diagnostic methods, exosomal biomarkers offer a non-invasive, cost-effective, and readily accessible routine screening through simple fluid collection that aims to inspire researchers to investigate the potential of exosomes for cancer theragnostic. Through comprehensive exploration of these areas, the review seeks to enhance understanding and foster innovative solutions to cancer biology in the near future.

## 1. Introduction

Bone cancer is a rare but serious form of cancer that develops in bone tissue. From young children to the elderly, it can afflict anyone. However, some types of bone cancer may be more prevalent in certain age groups. It is characterized by uncontrolled bone cell development. Bone cancer is mainly of two types: primary and secondary (metastatic) bone cancer. Primary bone cancer is comparatively rare and accounts for less than 1% of all the cancers diagnosed each year, but it has a significant morbidity and mortality rate. The most common primary bone cancer types are osteosarcomas, Ewing sarcoma, and chondrosarcoma 1, 2.

Osteosarcoma is the most common, accounting for about 31.5% of all cases of primary bone cancer, followed by chondrosarcoma and Ewing's sarcoma 3. Osteosarcoma is bimodal; it primarily affects children and adolescents, with the peak incidences occurring between 10 and 14 years of age. The second incidence usually occurs in adults above 40 years of age. However, it can also happen at any stage of life if a benign bone tumor transforms into a malignant one. In adolescents, it most frequently occurs in the metaphysis of long bones like the distal femur, proximal humerus, and proximal tibia, but it can also occur in other bones. In older patients, osteosarcoma develops mainly due to Paget's disease or irradiated osteolytic lesions 4, 5.

Osteosarcoma can be classified into 3 sub-groups, osteoblastic, chondroblastic, and fibroblastic, but they have no major differences 6. Ewing's sarcoma accounts for about 16% of all the bone sarcomas. It is most common in teenagers with a median age of 15, and its incidence rapidly decreases after 20 years. It typically develops in the diaphysis of long bones (proximal femur, proximal humerus, and proximal tibia) but can also occur in the pelvis, ribs, and scapula 6. The origin of the Ewing sarcoma cells is unknown. Recent studies have proposed that they develop from primitive stem cells and that the stage of stem cell arrest during differentiation determines the level of malignancy. Based on its microscopic characteristics, Ewing sarcoma is included in small blue round cell tumors 2, 7. Chondrosarcoma is most frequently diagnosed in patients above 40 years of age, and the rate of incidence gradually increases till 75 years. It constitutes about 25% of bone sarcomas. It can develop both as a primary tumor or as a benign bone tumor that has undergone malignant change. The most frequent sites of occurrence are the pelvis, ribs, scapula, proximal long bones, and vertebrae 4, 6. According to recent reports, these histologic subtypes, chondrosarcoma shows 30% occurrence in males and 29% in females; osteosarcoma shows 16% in males and 17% in females; Ewing's sarcoma around 14% in both males and females and chordoma 8% in males and 5% in females 8.

Secondary (metastatic) bone cancer occurs when cancer from another primary cancer site spreads to the bones. It is a severe complication in advanced disease; up to 70% of individuals with prostate cancer and breast cancer and up to 30% of those with lung, bladder, and thyroid cancer develop metastatic bone cancer 9. Several complex processes, including tumor-cell proliferation, cell-matrix separation, cell migration, angiogenesis, and intravasation, are involved in forming bone metastases at the initial location. The metastatic process is aided by the complicated capillary network and the slow blood flow in the bone marrow, where the tumor cells are transported via the vasculature or lymphatic system 10, 11.

**Figure 1:**
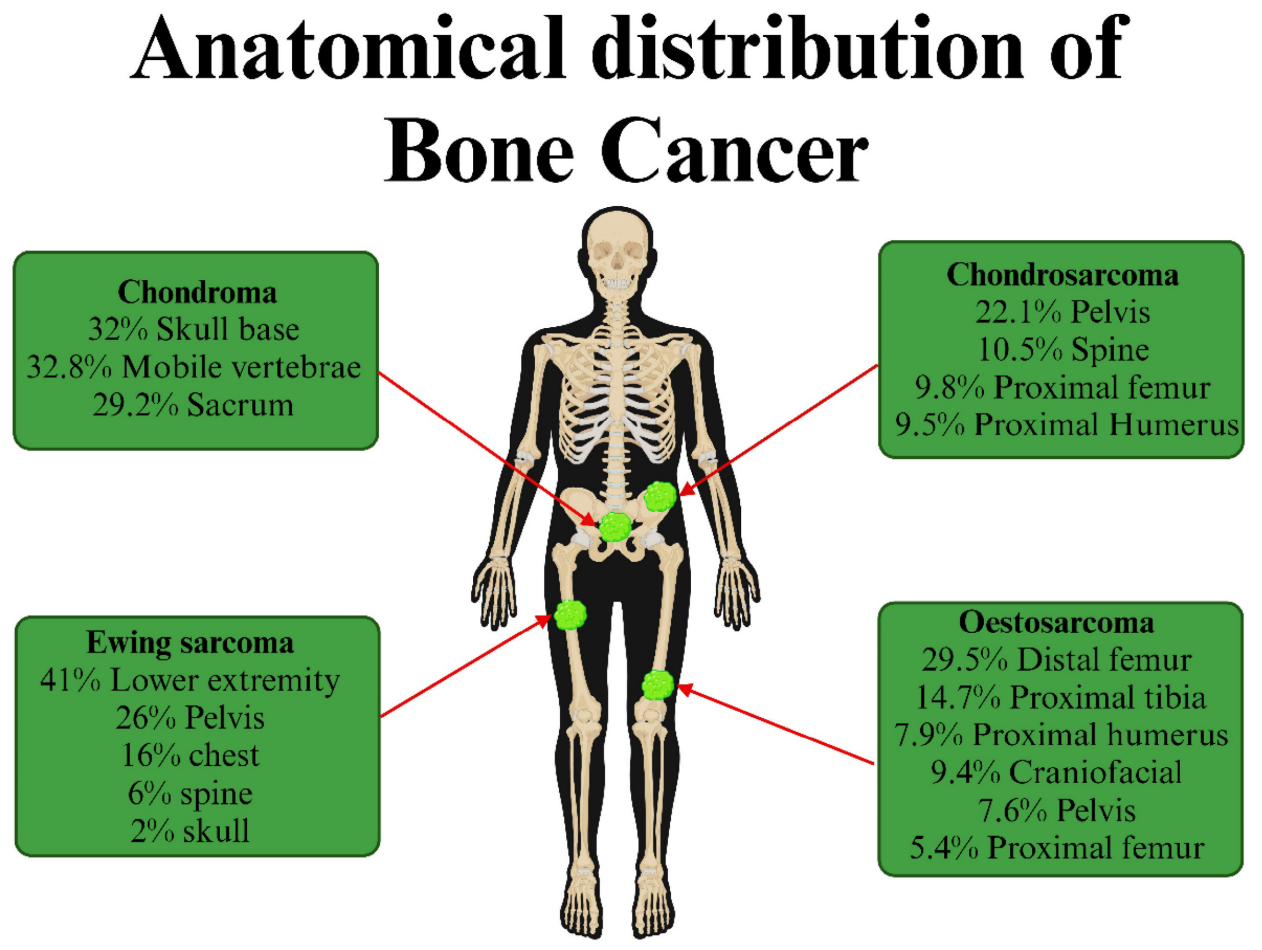
** Types of bone cancer.** Bone cancer can be classified based on the location of the tumor formed: Chondroma, osteosarcoma, Ewing sarcoma, and chondrosarcoma. (Created in BioRender.com).

**Figure 2:**
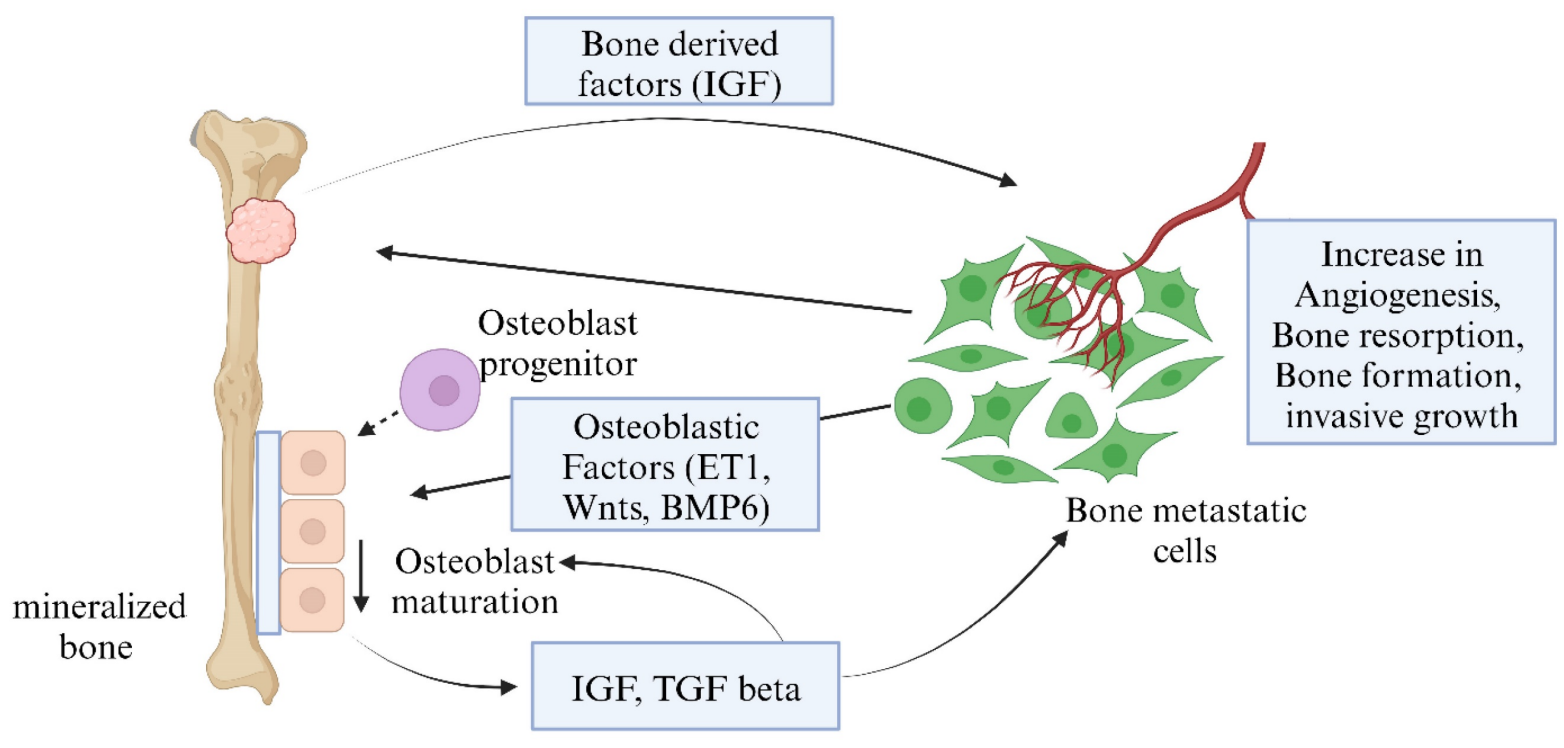
** Pathophysiology of osteosclerotic bone metastasis.** Osteoblast cells send various signals that are required for their maturation and metastasis. Bone metastatic cancer cells, with the help of these signals, can increase angiogenesis, bone resorption, and invasive growth (Created in BioRender.com).

Bone cancer is characterized by poor prognosis and high mortality since timely diagnosis is challenging due to late patient presentation, ambiguous symptoms that mimic common musculoskeletal ailments, and low suspicion by physicians. Traditional therapies (such as chemotherapy, radiation, and surgical resection) also face problems with drug resistance and disease recurrence, which restrict their application and effectiveness 12. In this sense, much effort must be put into finding and implementing improved bone cancer therapies. **Figure 1** shows the types and anatomical distributions of bone cancer. **Figure 2** shows the pathophysiology of osteosclerotic bone metastasis.

In detail, we have discussed bone cancer, its histological types, and anatomic distributions. Cancer research is evolving every day. Exosome research is becoming increasingly important, and we must look at their advantages and importance in cancer prognosis. Exosomes, small extracellular vesicles ranging from 30 to 150 nm in diameter, are essential in bone cancer research due to their integral roles in cellular communication and tumor microenvironment modulation. Characterized by a lipid bilayer membrane enriched with specific proteins such as tetraspanins (CD63, CD9, CD81), these vesicles originate from the endosomal compartment 13, 14. They are distinguished by their content of proteins, lipids, RNA, and DNA content, which aligns with criteria set by the International Society for Extracellular Vesicles 15. Exosomes facilitate tumor progression and metastasis by transferring oncogenic factors between cells, supporting angiogenesis, and modulating the immune response to favor tumor growth 16. They also contribute to drug resistance by mediating the transfer of resistance factors and drugs out of cells 17. Notably, the molecular signature of exosomes makes them excellent candidates for non-invasive biomarker discovery and promising vehicles for targeted drug delivery, revolutionizing diagnostics and therapeutics in bone cancer.

## 2. Biogenesis

**Figure 3:**
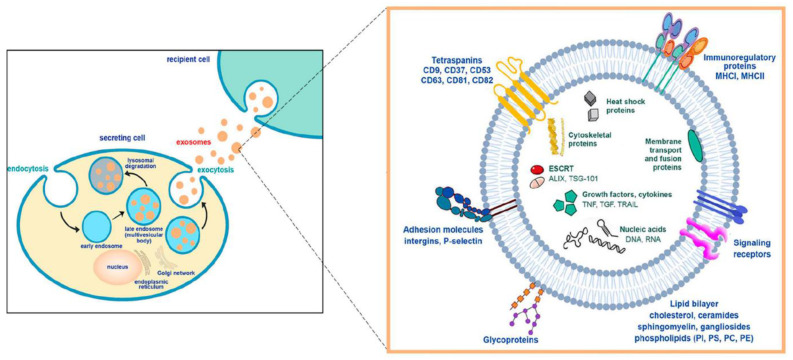
Exosome biogenesis and its molecular components (Reprinted with permission from ref 28 CC-BY 4.0 License Copyright © 2023 The Authors).

Exosomes, membrane-bound extracellular vesicles, are primarily generated within the endosomal compartment of most eukaryotic cells 18. These EVs are the intermediate by-products of plasma membrane-derived early- to late endosomes 12, 13. Processing of early endosomes (EEs) produces a subtype of endosomes carrying several membrane-bound intraluminal vesicles (ILVs) called multivesicular bodies (MVBs). These MVBs consequently fuse with the plasma membrane to release their contents outside cells to form exosomes 21. There are two distinct mechanisms by which exosomes are produced: ESCRT-dependent (Endosomal Sorting Complexes Needed for Transport-dependent) and ESCRT-independent 21. ILVs are produced by ESCRT using a sophisticated networking cascade 22 involving four types of complexes such as ESCRT-0, ESCRT-I, ESCRT-II, and ESCRT-III 23. In the initial stages of the pathway, ESCRT-0 binds to Zinc Finger Domains (ZFDs) and Ubiquitin-interacting Motifs (UIMs) 24, present in the plasma membrane through its dimeric subunits like hepatocyte growth factor regulated tyrosine kinase substrate (HRS) and signal-transducing adaptor molecule 1/2 (STAM-1/2) 25, 26. This subsequently activates ESCRT-I and ESCRT-II, which facilitates cytoplasmic budding from the plasma membrane guided by ESCRT-0, followed by mediation of cargo selection by ESCRT-II and ESCRT-III 19, 20. However, the scientific rationale for the ESCRT-independent pathway could be more evident. Ceramide-mediated membrane budding is linked to various cargo sorting and budding mechanisms 23. Because of their self-organizing ability and formation of a raft-like structure, they can enhance membrane budding during the biogenesis of exosomes 23** Figure 3**.

## 3. Exosome in immune suppression

The nanosized, membrane-bound extracellular vesicles of endosomal origin are known to be released by all cell types and are present in various body fluids. Now, the exosomes derived from tumors contain many functional cargos, including immunosuppressive molecules and other important factors that are known to disrupt standard immune cell functions 29. These tumor-derived exosomes (TEX), produced in abundance, are known to mediate the transfer of genomic DNA, mRNA, and even microRNA (miRNA) to the immune cells 27, 30. As a result, they end up reprogramming responder cell functions that promote tumor progression. It is observed that antitumor immunotherapies are disturbed by the tumor-associated antigens delivered by these TEX 31. They mediate biological effects like intercellular crosstalk or receptor discharge 32. First, immune system defense molecules restrict the progression of tumors. However, they are slowly blunted by TEX biomolecules known to activate immune suppression pathways.

The tumor microenvironment is known to play a major role in the development and progression of cancer 33. Even though they face resistance from the immune cells initially, tumor cells start molding the host environment such that it favors its expansion and proliferation. The release of immunosuppressive microvesicles or TEX mainly does this. The secretion of this TEX occurs spontaneously from the tumor cells. They are linked to some alterations in the function of the T cells of cancer patients 34. Changes include inducing apoptosis and variations in the T cell receptor functions and their component 35.

**Figure 4:**
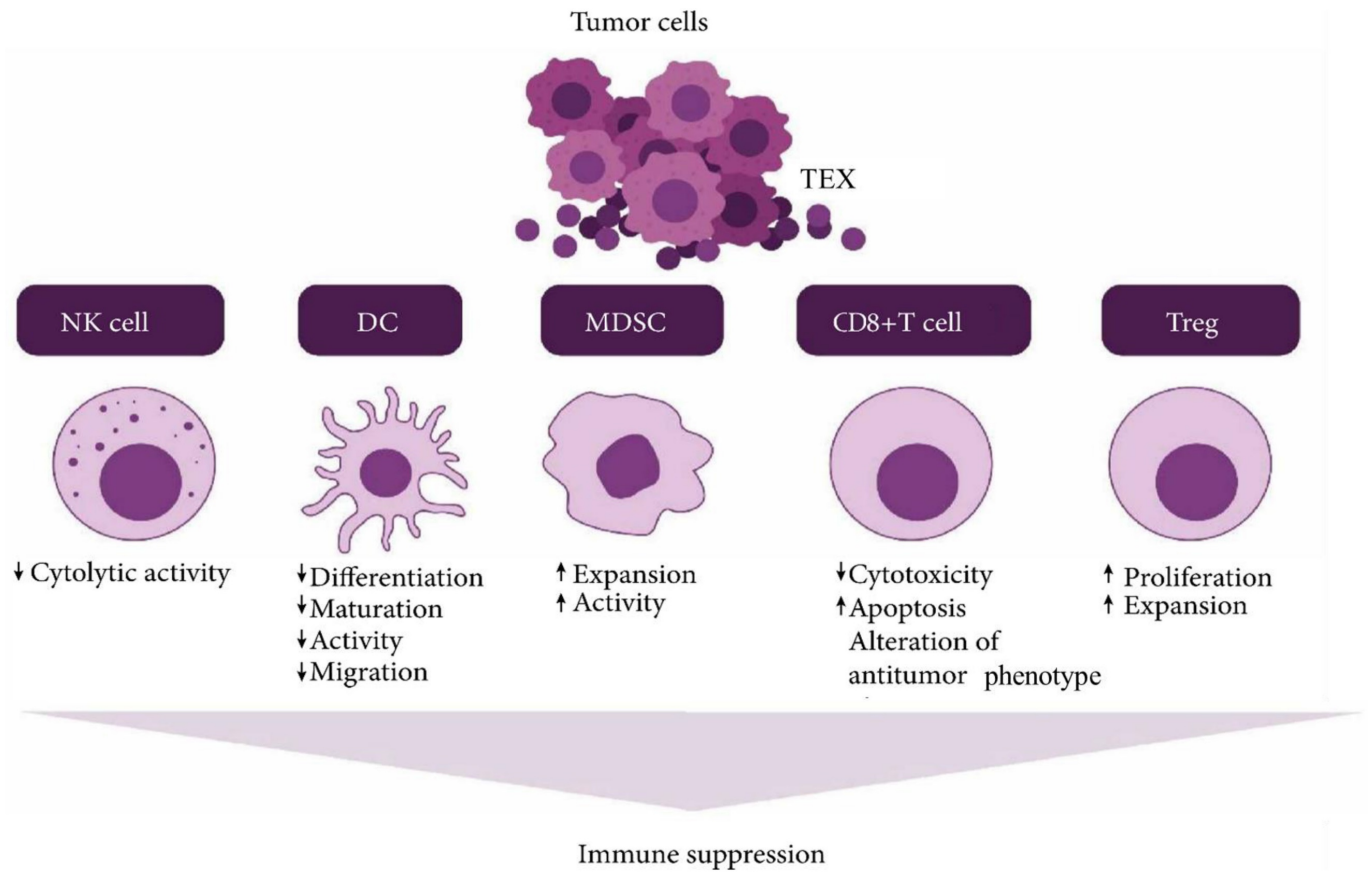
Tumor derived exosome (TEX) suppresses NK cell and dendritic cell (DC) activities, promotes myeloid-derived suppressor cell (MDSC) expansion, inhibits immune cell proliferation, induces apoptosis of activated CD8+ T cells, and promotes regulatory T cell (Treg) expansion (Created in BioRender.com).

Cancer immunosurveillance mediated by TEX is a process by which cancer maintains its immunity and stops the host immune cells from restraining cancer growth in the initial stages of cancer development 36. With the progression of the disease, tumor cells start adopting escape mechanisms by activating immunosuppressive pathways. These pathways are mainly conducted by cell-to-cell contact and by releasing suppressive factors that influence the differentiation of myeloids 37. TEX serves as an effective vehicle for conducting tumoral immunosuppression. They first interact with the host's immune cells with antigens or ligands that the lymphocyte receptors can recognize 38. Then, these exosomes fuse with the membrane and release their cargo into the cell's cytoplasm via receptor-mediated uptake. The phagocytic cells, especially the dendritic cells and macrophages, can rapidly internalize these exosomes. However, T cells do not take them up immediately; instead, it is done by the exosome's interaction with its surface molecules. This leads to signal generation and Ca^2+^ flux activating the downstream molecules that participate in signaling. Ultimately the change is observed in the transcriptome of the receiving cell 39. A summary diagram is provided in **Figure 4**. Linking the genetic and molecular profiles of these exosomes to the immunosuppressive effects in cancer is still being monitored and is in progress.

## 4. Angiogenesis

Angiogenesis is the formation of new blood vessels predominantly governed by various growth factors and cytokines. This mechanism plays an important role in cancer progression. Hypoxic tumor microenvironment induces the expression of Hypoxic inducible factors (HIFs), which sends a triggered signal for angiogenesis in lieu of enhanced pro-angiogenic factor expression like vascular endothelial growth factor (VEGF), Tumor necrosis factor-α (TNF-α) 40,33. These pro-angiogenic factors enhance angiogenesis via endothelial cell sprouting 41 or recruit endothelial progenitor cells (EPCs) from bone marrow cells. In Ewing sarcoma, the endothelial cells exhibit an enhanced proliferative rate in the presence of VEGF 42. This growth factor plays a key role in angiogenesis. Angiogenesis can also be enhanced in the presence of insulin growth factor-1 (IGF-1), which directly controls the expression levels of VEGF. Anti-angiogenic factor downregulation also increases the probability of angiogenesis. Like in Ewing sarcoma, angiogenesis can be reduced by thrombospondins, an anti-angiogenetic factor 43. Apart from the hypoxic microenvironment, mechanical stress, genetic mutations, and inflammatory responses can also lead to angiogenesis 44. Increased vascular density (VD), an important characteristic for primary bone tumors (osteosarcoma), can also indicate angiogenesis. Therefore, angiogenesis promotes tumor growth and causes bone destruction via enhanced osteoclastic response, disturbing bone remodeling balance 31, 43. Sometimes, angiogenesis-associated factors like matrix metalloproteinases (MMPs) degrade bone matrix components, disrupting the overall structural integrity of bone. Thus, it is very important to understand the molecular mechanisms of angiogenesis, and targeting pro-angiogenic factors can be a potential target for targeted therapy to treat osteosarcoma.

Exosomes derived from cancer cells have been shown to carry pro-angiogenic factors, such as VEGF and fibroblast growth factor (FGF). When these exosomes are taken up by endothelial cells, which line the interior of blood vessels, they can stimulate angiogenesis by promoting the proliferation, migration, and tube formation of these endothelial cells. In bone cancer, where angiogenesis can be a critical factor in tumor progression and metastasis, the release of pro-angiogenic exosomes may contribute to the development of new blood vessels within the bone tissue. This, in turn, can support the growth and spread of cancer cells within the bone 38, 39, 40.

## 5. Metastasis (ECM, EMT, Organ-specific)

Among different types of cancers, osteosarcoma is highlighted for having a rich extracellular matrix (ECM) composition as a hallmark for metastasis 49. ECM consists of a 3D acellular framework of macromolecules, which is required to provide mechanical strength to a cell's cellular components 50. It also controls cellular activities like adhesion, migration, proliferation, etc. 51. It has been reported that remodeling of the ECM components in osteosarcoma results in the progression of invasion and metastasis (Refer to **Table 1)**. MMPs and Heparinases are the vital regulatory players for osteosarcoma proliferation. Upregulation of several MMPs (MMP-2, MMP-9, MMP-13) via P13K/Akt and ERK pathways leads to the degradation of ECM components and the production of tumor progression-inducing active growth factors 52. Besides, Heparinase (endo-β-D-glucuronidase) promotes metastasis of osteosarcoma by cleaving heparan sulfate chains to release angiogenic components 53. Therefore, targeting ECM components and signaling players can be a potential therapeutic approach.

Another factor for metastasis, epithelial to mesenchymal transition (EMT), is a transformation phenomenon where the epithelial cells lose their markers, apical polarity, and adherence capacity to become mesenchymal cells. Regulation of EMT is complex and affected by several upregulated transcription factors, like Snail 46, 47, Slug, ZEB1 36, and Twist 56 and different pathways (*wnt*/β-catenin, Notch, Tyrosine Kinase, transforming growth factor-β (TGF-β) pathways, etc.) that crosstalk with each other. Recent studies have shown that TGF-β ligands promote the phosphorylation of SMAD proteins (SMAD2, SMAD3) to make a complex with SMAD4 that activates Snail, Slug, and ZEB1 while repressing E-Cadherin for a positive feedback loop of EMT metastasis. Moreover, the Canonical* wnt*/β-catenin pathway, like the Notch pathway, upregulates the expression of Twist, Slug, and N-Catherin, where it suppresses the E-Catherin. Researchers have reported that the epidermal growth factor (EGF) binds to its receptor to downregulate E-Cadherin via activating the MAPK pathway. The JAK/STAT pathway also promotes EMT using Twist. Besides, inhibition of apoptosis by phosphorylated Twist1 is another EMT-promoting mechanism of the Akt pathway. Human telomerase reverse transcriptase promoter (hTERT), long non-coding RNAs (LncRNAs), circular RNAs, and miRNAs are vital for EMT-induced metastasis 49, 50. Further investigation showed that the osteosarcoma microenvironments, tumor-associated macrophages (TAMs), and overexpressed cyclo-oxygenase 2 (COX2) are also EMT-responsible players 59. Additionally, transmembrane protein (Tspan9) interacts with integrin B1 to enhance the EMT-inducing FAK-RAS-ERK1/2 axis for promoting osteosarcoma metastasis 60, 61. These transcriptional factors and their crosstalk can be used as the target site of therapies.

ECM, EMT degradation, and alteration lead to the outcome of osteosarcoma metastasis in specific organs. The major metastatic site for osteosarcoma is the lungs, though few cases of liver and brain metastasis are enlisted 63. Organ-specific metastasis shows three stages: A) Spreading of osteosarcoma cells from the primary site, which involves activated osteoblasts to produce receptor activator of NF-κB ligand (RANKL) to interact with the bone-resorption inducing osteoclasts (OCs). Various researchers have stated that several MMPs (MMP-9, MMP-13, MMP-14, MMP-16) and cathepsin are critical regulators of organ-specific metastasis. Moreover, the binding between osteosarcoma-secreted stromal cell-derived factor 1 (SDF1) and mesenchymal Stem cells (MSCs)-induced CCL5 promotes this metastasis 64. During the metastasis, NK cells can kill the osteosarcoma cells via natural killer group 2 member D (NFK2D)-ligand (NFK2DL) binding 65. B) Spreading through blood vessels where urokinase-type plasminogen activator (uPAR), runt-related transcription factor 2 (RUNX2), osteopontin (OPN), and formyl peptide receptor type-1 (FPR-1) help the osteosarcoma cells to invade the blood vessels, resulting in metastasis. Here, fatty acid synthase (FSN) and inhibitor of DNA breaking (ID1) induce adherence of osteosarcoma cells to endothelial cells in the lung to create resistance against potential therapies 64. C) Colonization in specific organs involves glucose-related protein 78kD (GRP78) and activating transcription factor 6 (ATF6) to avoid apoptosis via the NF-κB pathway. Some tumor cells produce extracellular vesicles, specifically exosomes, to interact with the resident cells to prepare a pre-cancerous environment in the organ 66. Recent advancements in site-specific metastasis showed that CXCL1/CXCL2 axis is also a good therapeutic target for lung metastasis in osteosarcoma 67.

**Figure 5:**
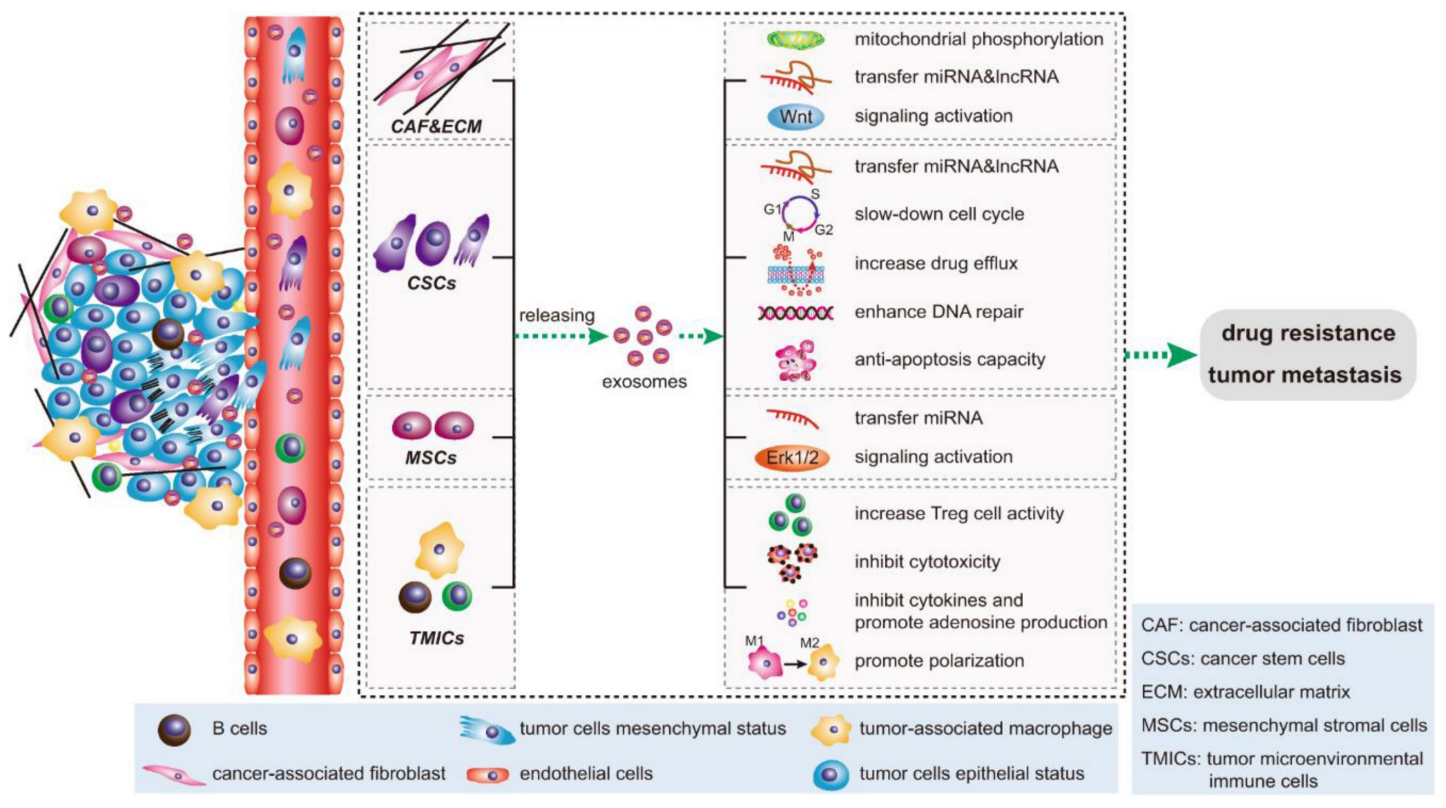
Signal transduction pathways of exosomes in the tumor microenvironment. Key cellular components within the tumor microenvironment, notably cancer-associated fibroblasts (CAFs), cancer stem cells (CSCs), mesenchymal stromal cells (MSCs), and tumor microenvironmental immune cells (TMICs), utilize exosomes to facilitate epithelial-mesenchymal transition (EMT), enhance tumor metastasis, and foster drug resistance through diverse mechanisms. (Reprinted with permission from ref.^62^ CC-BY 4.0 License ©2020, The Author's).

Bone cancer cells may release exosomes containing various bioactive molecules. These exosomes can serve as messengers facilitating communication between cancer cells and the surrounding bone tissue or other distant sites. They deliver enzymes like matrix metalloproteinases (MMPs), influencing ECM degradation and modification and facilitating processes such as cell migration and invasion. Additionally, exosomes transfer bioactive molecules to recipient cells, affecting cellular behavior in response to ECM stiffness. Exosomes promote a supportive tissue repair and regeneration microenvironment by carrying growth factors and cytokines. Moreover, exosomes mediate communication between cells and the ECM, providing crucial insights into tissue homeostasis and disease processes. Exosomes can carry specific cargoes of proteins, nucleic acids, and lipids that influence various steps of the metastatic cascade, including invasion, migration, immune evasion, and establishing secondary tumors in the bone or other organs. One critical aspect of bone metastasis is the interaction between cancer cells and the bone microenvironment. Cancer cells release exosomes can contain factors promoting bone degradation, such as MMPs and RANKL. These factors can stimulate the activity of osteoclasts, cells responsible for breaking down bone tissue. As a result, the bone matrix is altered, creating a favorable niche for cancer cell colonization and growth. Additionally, exosomes may carry molecules that modulate immune responses, allowing cancer cells to evade detection by the immune system. This immune suppression can further facilitate the survival and growth of cancer cells at metastatic sites in the bone 38, 58. **Figure 5** represent the overall signal transduction pathways of exosomes in the tumor microenvironment and drug resistant.

## 6. Drug and therapeutic resistance

Bone sarcomas predominantly exhibit their heterogeneous nature within the tumor cells, which exhibit properties similar to stem cells. Hence, these heterogenous cells are called cancer stem cells (CSCs) 12. They may have originated from MSCs 73. These cells have a very high drug resistance ability to conventional drugs like methotrexate, doxorubicin, cisplatin, etc. When these drugs are administered to patients, CSCs activate the *wnt*/β--catenin signaling pathway, directly promoting the drug resistance property 12. Chemo-resistant phenotype is also governed by epigenetic modifications, activation of detoxifying pathways 74, and very high DNA repair capability 67. The tumor microenvironment plays a pivotal role in this phenotype. Low oxygen or hypoxic microenvironment not only enhances drug resistance ability but also escalates CSC formation via the expression of HIF-1α 75. It has also been seen that the* wnt/β-*catenin pathway facilitates hypoxia-induced drug resistance in bone sarcoma 76. Conventional therapeutic methods have failed to treat sarcomas in the long run because, in most cases, the recurrence of tumors or minimal destruction of CSCs takes place 77. Thus, recent advancements have been made in treating bone sarcomas via targeted therapies which are believed to be more specific than conventional treatments 78. Many new targets have been identified for bone cancer treatment, which will help develop new therapeutic strategies to overcome the drawbacks of conventional drug therapies. Glycoprotein A repetitions predominant (GARP) can serve as a potential target for treating bone sarcomas via the activation of TGF-β, but the main shortcoming of this potential target is it is not exclusively present in cancer cells; it is also present in normal tissues 79. Also, therapies targeting IGF-1R have shown very promising results in pre-clinical studies. However, the IGF-R1 inhibitors promote receptor downregulation, resulting in reduced IGF activity, which might, in turn, increase angiogenesis and tumor growth 80. Hence, targeting various downstream pathways and the IGF system is essential. Also, therapies targeting mTOR signaling pathways 72, inhibitory compounds targeting cell cycle progression 73, and apoptosis have been developed recently, which are still under clinical trial phases. Hence, multiple therapies can be clubbed together to target multiple aspects of the tumor cells for a successful bone cancer treatment. Hence, along with discovering various new targets, it is important to develop an accurate pre-clinical model and understand the underlying molecular mechanisms of osteosarcoma development.

Cancer cells within the bone tumor can release exosomes containing factors that promote drug resistance. These factors may include drug-efflux pumps, which actively pump chemotherapy drugs out of cancer cells, reducing their effectiveness. Exosomes can transfer these pumps to neighboring cancer cells, conferring resistance to a broader population of cells. Exosomes released by bone cancer cells can interact with the bone microenvironment, including osteoblasts and osteoclasts. This communication may trigger signaling pathways that enhance cancer cell survival and resistance to therapy. For example, exosomes can promote osteoclast activation, which leads to bone resorption and further supports cancer cell growth. The imbalance in the RANKL-RANK-OPG system, influenced by exosomal cargo, favors osteoclast activation. This process remodels the bone microenvironment and releases growth factors stored in the bone matrix, supporting cancer cell survival and proliferation. Additionally, exosomes may contain matrix metalloproteinases contributing to extracellular matrix remodeling and angiogenic factors, further facilitating cancer cell invasion and the formation of a vascularized niche. Exosomes can carry molecules that suppress the immune system's ability to recognize and attack cancer cells. This immunosuppressive effect can indirectly contribute to resistance by allowing cancer cells to evade immune-mediated destruction, even in the presence of therapies that stimulate immune responses 72, 73.

## 7. Biomarkers for OS

**Figure 6:**
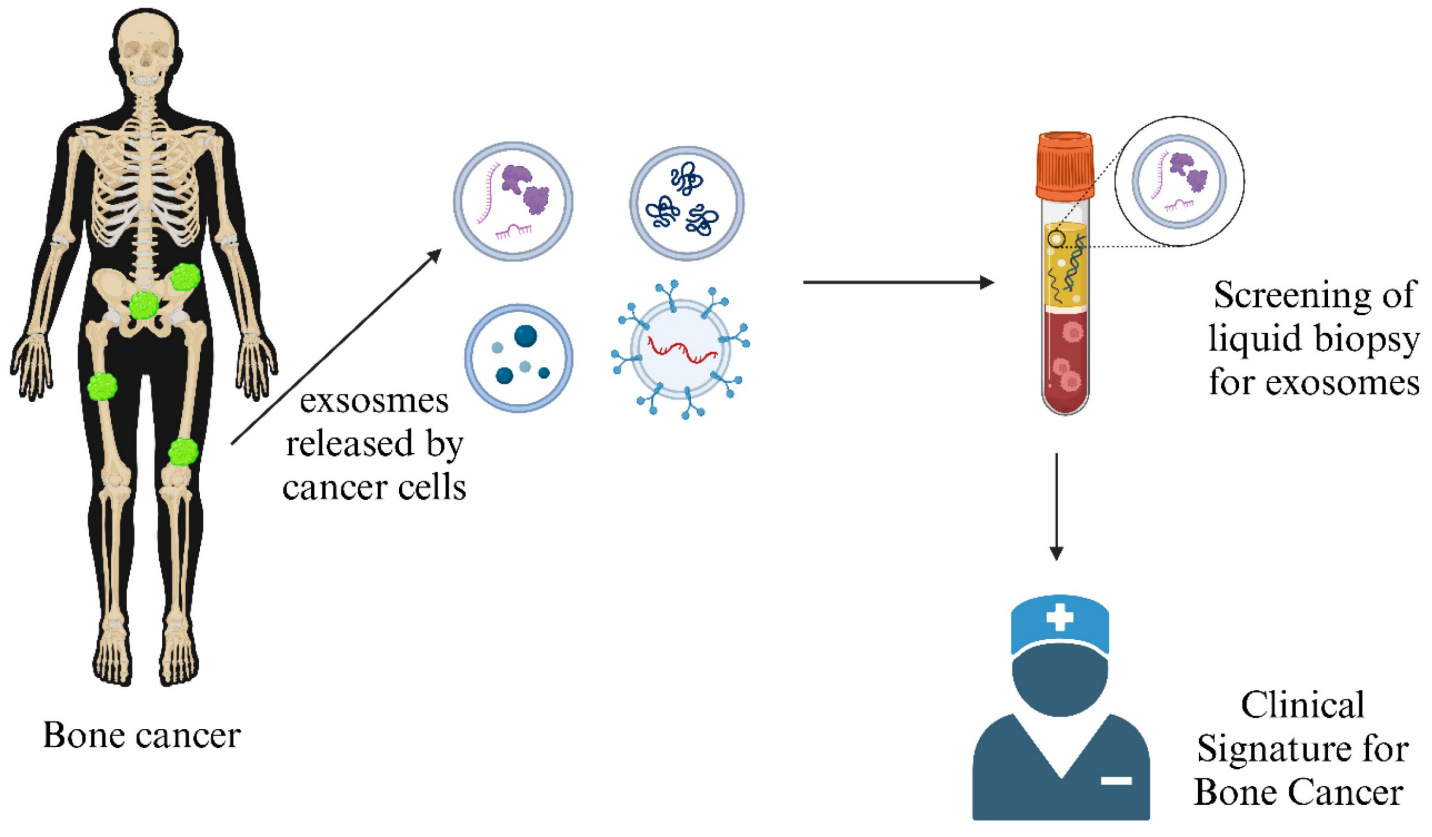
** Exosome as a Clinical Signature of Bone Cancer:** Exosomes secreted by bone cancer cells often carry unique cargo, such as specific proteins and genetic material, which can serve as biomarkers for the presence and progression of bone cancer. (Created in BioRender.com).

Various cancers, such as osteosarcomas, require early diagnosis to detect tumors before onset to prevent malignancies and invasiveness. Two types of biomarkers are available to check the probability of occurrence of a disease. Diagnostic markers help to detect tumor presence, whereas prognostic markers help study the chance of being tumor-positive. Liquid biopsy involving bodily fluids like blood, serum, and urine is a non-invasive, advantageous technique **Figure 6**. It helps in quantifying circulating tumor cells (CTCs), dissented tumor cells (DTCs), circulating tumor DNA (ctDNA), circulating miRNAs, LncRNAs, enzymes, immune cells, and exosomes from a patient's blood sample 83. Genetic mutation profiles for osteosarcoma can be studied by analyzing these markers. It has been reported that mutations in tumor-suppressing genes (TP53 and RB1) are prominent in cancer cells compared to normal cells, which act as prognostic markers for osteosarcoma detection 84. Further gene expression studies revealed that RUNX2, CDC5L, MDM5, RECQL4, and CDK4 can also be predictive biomarkers, although the mechanism remains unclear 85.

A potential biomarker candidate is miRNA. Several miRNAs like miR-195, miR-99, miR-135b, miR-150, miR-542-5p, miR-148a, etc., are upregulated in the serum of patients having bone sarcoma. In contrast, some miRNAs are downregulated in osteosarcoma cells than the normal osteoblasts, such as miR-34b, miR-539, miR-145, etc. 75, 77.

About 21 immune cells, such as MSC-secreted interleukin-6 (IL-6), TGF-β, hepatocytic growth factors, MMPs, etc., are overexpressed in osteosarcoma cells 87. Pro-peptides, like β-isomerized C-terminal telopeptides (β-CTX) and total pro-collagen type-1 amino-terminal pro-peptide (tP1NP/NTX), are also similarly overexpressed 88. Moreover, cellular hypoxic response triggers factors, such as HIF-1α, apurinic/apyrimidinic endonuclease 1 (APE1), VEGF, and COX2, are responsible for tumor development and metastasis 85. LDH and ALP enzymes are the diagnostic biomarkers for prognosis 89. Recent studies also revealed that Erizin, the linker agent between the plasma membrane and cytoskeleton, also shows overexpression in osteosarcoma cells compared to normal cells 90. **Table 2** lists some exosome biomarkers, their sources, and the expression profile for osteosarcoma prognosis.

Exosomes, tiny vesicles released by cells, have gained attention as potential biomarkers in various cancers, including bone cancer. These vesicles contain a cargo of proteins, nucleic acids, and lipids that reflect the status and characteristics of the cells from which they originate. Exosomes in the bloodstream can carry specific proteins or genetic material originating from bone cancer cells. Detecting these unique markers in circulating exosomes may enable the early diagnosis of bone cancer, even before clinical symptoms or visible tumors appear. Exosome analysis may provide valuable prognostic information, helping clinicians predict the likely course of the disease and overall patient outcomes. Exosomes can offer insights into how bone cancer cells respond to treatment. By analyzing the contents of exosomes, clinicians can determine whether cancer cells are becoming resistant to therapy, allowing for timely adjustments in treatment plans 91.

## 8. Therapeutic Approach

Treatment of bone cancer depends on several factors, such as the type and characteristics of the cancer, whether the disease is localized or extensive, the presence of extra-skeletal metastases, the patient's symptoms, the history of previous treatments and the general health of the patient all play an essential role in the choice of treatment 102. The most popular method for treating bone cancer (malignant tumors) is surgical resection with appropriate margins in conjunction with radiation and/or chemotherapy 94, 95, 96.

However, to reduce the risk of local recurrence in malignant tumors, the surgical margins must be wide or radical, which requires that a layer of normal tissue cover all resected tumors. The treatment protocol includes surgical resection of the primary bone tumor as well as bone metastasis. The subtype of the disease further defines the surgical margin, the reconstruction, and the adjuvant therapy plan. The treatment plan for metastatic bone diseases is patient-specific and is chosen by the surgeon depending on the remaining predicted life expectancy of the patient 106. In unresectable or incompletely resected tumors, radiation was found to be useful as adjuvant therapy for easing localized bone discomfort and inflammation. Radiation therapy can be used successfully as a palliative intervention to preserve and enhance a patient's quality of life, lessen the need for analgesics, and improve skeletal function 107. It may also be used to treat Ewing sarcoma, but chondrosarcomas are considered radioresistant; hence, other methods like ablative approaches must be used as substitutes or in combination for their treatment 102, 108. Chemotherapy is another method commonly used for the treatment of sarcomas. Neoadjuvant chemotherapy regimens are used to induce tumor necrosis, lower the size of the main tumor, and lessen the quantity and size of lung metastases 2. Doxorubicin, cisplatin, high-dose methotrexate, and ifosfamide are some current chemotherapeutic options 108. These drugs act against cancer by disrupting one or more cell cycle steps. They are administered in treatment cycles, and their schedule depends on the healthy tissues' healing ability 109. However, due to its multidrug resistance, chemotherapy is ineffective against chondrosarcomas 110. The treatment often shrinks the size of the tumors or slows the progression of bone metastases, relieving symptoms, but it is not always curative. After the initial response to treatment, tumors might return and behave more aggressively while exhibiting increased resistance to systemic treatments 102, 111. These existing methods of studying bone cancer have several obstacles. To increase survival rates and prevent illness recurrence, it is important to identify better and modern treatment approaches and overcome the drawbacks of conventional therapies either by replacing them or working in tandem.

These new approaches include targeted therapy and radionuclide therapy (such as Xofigo). Combination therapy refers to combining two or more therapeutic methods like chemotherapy, radiotherapy, immunotherapy, and hormone therapy or simultaneously delivering two or more therapeutic agents. The most popular type of combination therapy for treating cancer in clinical practice is the co-delivery of different chemotherapeutic drugs 3, 112. Combination therapy aims to boost the effectiveness of treating cancer while reducing systemic toxicity and combating drug resistance 113. Nanoparticles have also become extremely prevalent in drug delivery for cancer therapy, particularly combinatorial therapy 112, 114. The method for enhancing chemotherapy has radically changed due to developing better transport systems using nanotechnology. By co-delivering various payloads, enhancing biodistribution and transport qualities, normalizing accumulation, and optimizing release profiles, nanotechnology has helped to progress cancer therapy 115. Similarly, adjuvant therapy decreases the risk of local recurrence and prevents the need for extensive resection and reconstruction 102, 116. Radiofrequency, cryoablation, laser, and microwave ablation are common and widespread adjuvant therapeutic methods. Recent studies have identified new molecular targets within bone cancer cells. Targeted therapies like monoclonal antibodies and tyrosine kinase inhibitors are made specifically to inhibit these abnormal pathways. Tissue engineering approaches have also shown promising results in treating bone sarcomas. Tissue engineering enables the development of intelligent, multifunctional bone substitutes by using a porous structure (called scaffold) as a template for cell attachment, differentiation, proliferation, and tissue regeneration 103, 117. Synthetic bone substitutes have emerged as a promising replacement for autografts, allografts, and xenografts over the past ten years 118. It is also found that patients are more likely to accept the use of these synthetic bone grafts for surgical operations 119.

**Figure 7:**
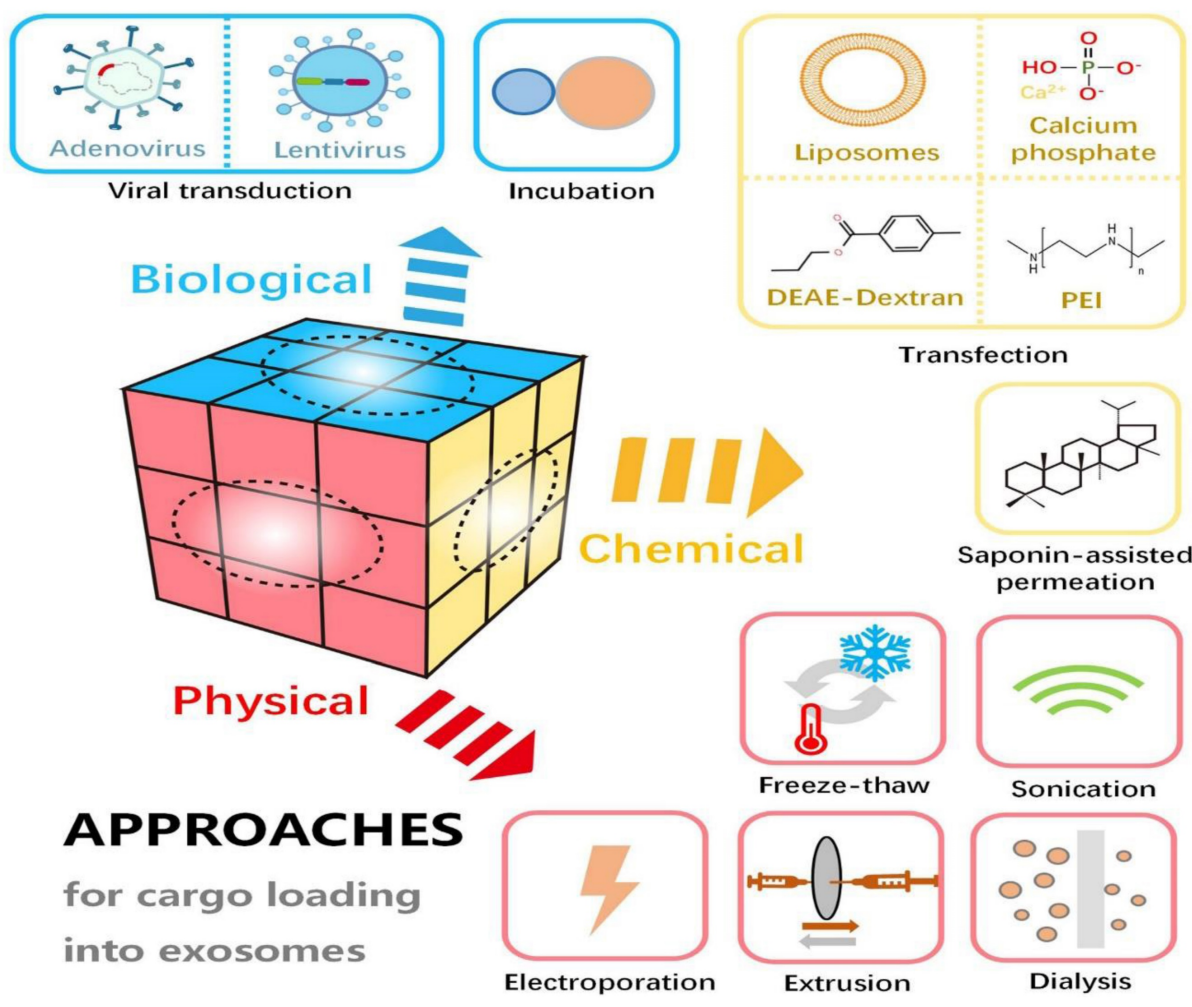
Exosome loading approaches. (Reproduced with permission under Creative Commons CC BY 4.0 license from ref. 121 © 2021 Frontiers Media.) [Abbreviations: DEAE-dextran, diethylaminoethyl-dextran; PEI, polyethylenimine.].

Exosomes serve as effective vehicles for transporting therapeutic agents, including miRNA and pharmaceuticals. Focusing on exosome-based systems for drug delivery overcomes many constraints associated with conventional methods. These natural vesicles offer several advantageous properties, such as high biocompatibility, minimal immunogenicity, reduced toxicity, enhanced target specificity, and the capacity to traverse biological membranes 120. Various techniques are employed to load therapeutic payloads into exosomes. A few methods used for cargo loading are summarized in **Figure 7**.

The therapeutic landscape for bone cancer is rapidly evolving, with innovative approaches offering hope for improved outcomes and enhanced patient well-being. The recent advancements ranging from adjuvant therapy to precision medicine and tissue engineering to gene editing are encouraging. As researchers and clinicians continue collaborating and innovating, the future holds promise for more effective and less invasive treatments for this challenging disease.

## 9. Clinical Trials

Clinical trials play a crucial role in advancing our understanding of cancer. In recent years, researchers have turned their attention to the potential of exosomes in the context of bone cancer. Exosomes, small vesicles released by cells, contain RNA and proteins that may serve as valuable biomarkers and therapeutic targets. **Table 3** highlights three distinct clinical trials focused on exosome research in bone cancer. These trials aim to harness the power of circulating exosomes to improve early diagnosis, prognosis, and treatment monitoring in patients with bone cancer, particularly osteosarcoma.

## 10. Future Prospects

Cancer remains one of the top causes of death globally. Despite considerable progress in medical science and a deeper understanding of cancer's molecular mechanisms, along with advances in detecting and treating the disease, cancer incidence rates continue to be substantial, and a definitive cure has yet to be found. One of the most exciting directions involves the refinement of exosome-based biomarkers for early detection and monitoring of bone cancer. As our understanding of the specific cargo carried by bone cancer-derived exosomes deepens, we anticipate the development of highly sensitive and specific diagnostic tests. Moreover, exploring exosome-based therapies, including the engineering of exosomes for targeted drug delivery, holds great potential in overcoming the challenges of drug resistance and enhancing treatment efficacy. Continued investigation into the crosstalk between exosomes and the immune system is poised to unlock novel immunotherapeutic strategies, including developing exosome-based vaccines and immunomodulatory interventions. Harnessing the full potential of exosomes as mediators of immune suppression and disease progression may pave the way for innovative therapeutic approaches that can transform the landscape of bone cancer management in the years to come.

## 11. Conclusion

This review has highlighted the multifaceted nature of bone cancer and its complex interactions within the tumor microenvironment. While much progress has been made in understanding its various aspects, the future of bone cancer research lies in unraveling the enigmatic role of exosomes. Exosomes are emerging continuously in bone cancer management due to their unique properties and advantages over traditional diagnostic and therapeutic methods. These naturally derived vesicles are highly biocompatible and exhibit minimal immunogenicity, ensuring safety and reducing adverse reactions in clinical applications. One of the standout features of exosomes is their enhanced targeting capabilities. They possess natural homing abilities to specific cells, which can be engineered for increased specificity towards bone cancer cells, thus improving therapeutic efficacy and minimizing off-target effects. Furthermore, exosomes can traverse challenging biological barriers, including the blood-brain barrier, making them invaluable for treating hard-to-reach metastatic cancers. Their ability to carry diverse therapeutic agents—from small molecules to RNA and proteins—offers versatility in delivering multiple therapies, including emerging gene therapies.

In diagnostics, exosomes are non-invasive biomarkers, enabling real-time disease progression and treatment response monitoring through simple blood samples. This aspect is crucial for personalized medicine, allowing therapies to be tailored based on the tumor's molecular profile reflected in exosome contents, thereby optimizing treatment outcomes with minimal toxicity. The potential of exosomes to revolutionize bone cancer prognosis and therapy positions them as a focal point of interest for clinical researchers, promising a new era of enhanced patient management and treatment strategies. These tiny vesicles are pivotal in immune suppression, angiogenesis, metastasis, drug resistance, and biomarker discovery. As we delve into the intricate world of exosomes, novel diagnostic and therapeutic avenues will likely emerge. By harnessing the potential of exosomes, we hope to transform bone cancer management, ultimately improving patient outcomes and providing new hope for those affected by this devastating disease.

## Figures and Tables

**Table 1:** List of the ECM components, their activities, and their level of expression in the osteosarcoma environment

ECM components	Activities	Expression level	References
Collagens (I, III, IV, V, and XVIII)	Invasion Adhesion Metastasis Resistance against therapies	Over-expressed, except Collagen XVIII, which gets downregulated	59, 60
Laminin	Invasion Adhesion	Over-expressed	71
Fibronectin	Invasion Adhesion Metastasis Drug resistance	Over-expressed	72
Proteoglycans (Biglycan, Lumican, Decorin, Hyaluronic Acid, etc.)	Invasion Metastasis Migration Apoptosis	Over-expressed except Decorin (downregulated)	49

ECM: extracellular matrix

**Table 2:** Exosome-based Bone Cancer Biomarkers

Types of Biomarkers	Names	Exosome Sources	Expression profile	References
Prognostic	TP53 gene	ctDNA from blood, serum	Mutations and altered P13K/mTOR pathway in OS patients	84
	RB1 gene			
	MMP9	CTC analysis from blood	Overexpressed in OS patients	92
	HIF-1α	CTC analysis from blood	Upregulation leads to a lower survival rate	75, 84
	VEGF	CTC of Patient's blood	Overexpression in OS female patients	
	APE1	Serum	Upregulation results in adverse prognosis	94
	CTX	Serum, Urine	Upregulated	86, 87
	NTX			
Diagnostic	TIM3	NK cell membrane expressed isolated from blood CTCs	Overexpressed in OS patients.	85
	TGFβ	Serum	Upregulated	87
	Galectin 3	Serum	Upregulation in OS tissues and cell lines related to adverse prognosis	97
	Lactose Dehydrogenase	Serum, Blood	High level is OS patients.	98
	Alkaline Phosphatase	Serum, Blood	High level leads to poor prognosis	99
Diagnostic and Prognostic	Ezrin gene	CTC analysis from a blood sample	Upregulation leads to poor prognosis	90
	Src	ctDNA from blood	Overexpressed in OS patients	100
	miRNAs (miR-1908, miR-9, miR-195-5, miR-199a-3p, miR-320a, miR-374-5p, miR-148, miR-135b, miR-150, miR-542-5p)	Blood, serum	Upregulated in OS cell lines and patients	101
	miRNAs (miR-133a, miR-193a-3p, miR-193a-5p, miR-452, miR-206, miR-126, miR-199a-3p, miR-223, miR-34b, miR-539, miR-145)	Blood, Serum	Downregulated in OS cell lines and patients	

TP53 gene - Tumor Protein 53 gene; RB1 gene - Retinoblastoma 1 gene; MMP9 - Matrix Metallopeptidase 9; HIF-1α - Hypoxia-Inducible Factor 1-alpha; VEGF - Vascular Endothelial Growth Factor; APE1 - Apurinic/Apyrimidinic Endonuclease 1; CTX - C-terminal Telopeptide of Type I Collagen; NTX - N-terminal Telopeptide of Type I Collagen; TIM3 - T-cell Immunoglobulin and Mucin Domain 3; TGFβ - Transforming Growth Factor beta; Galectin 3 - Galectin-3; LDH - Lactate Dehydrogenase; ALP - Alkaline Phosphatase; Ezrin gene - Ezrin gene and Src - Proto-oncogene Tyrosine Kinase Src.

**Table 3:** Clinical Trials of Exosomes in Bone Cancer

Clinical trial ID	Sponsor	Responsible party	Summary
NCT03108677	Ruijin Hospital	Yuhui Shen, Ruijin Hospital	To investigate the use of circulating exosome RNA profiles as biomarkers for lung metastases in primary high-grade osteosarcoma. Researchers have found significant differences in circulating exosome RNA levels and mutations between patients with and without lung metastasis.
NCT05101655	Ruijin Hospital	Ruijin Hospital	The development of exosome microfluidic chips for early diagnosis of osteosarcoma lung recurrence and monitoring the response to second-line therapy for recurrent osteosarcoma. The microfluidic chip technology aims to capture and quantify exosomes for biomarker screening.
NCT03895216	Istituto Ortopedico Rizzoli	Istituto Ortopedico Rizzoli	To identify deregulated miRNAs within circulating exosomes in oncological patients with bone metastases. The goal is to establish a panel of predictive biomarkers for bone metastasis risk and explore their therapeutic implications. The study involves various molecular screening methods and 3D culture experiments.
